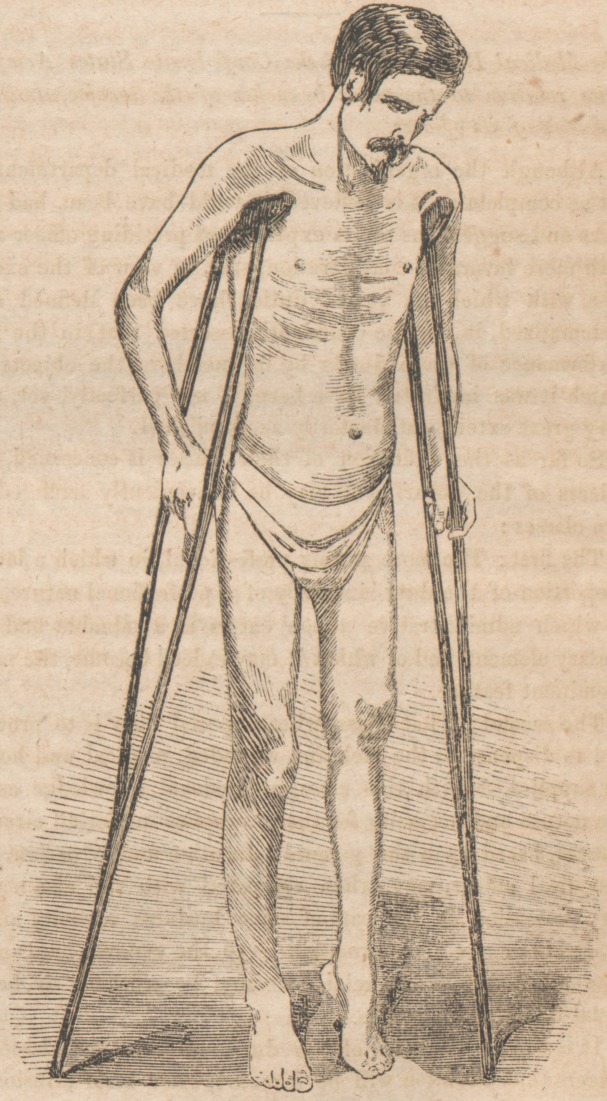# Editorial and Miscellaneous

**Published:** 1864-02

**Authors:** 


C. S. Medical & Surgical Journal.

RICHMOND, FEBRUARY, 1864.

*AYRES & WADE.....PUBLISHERS AND PROPRIETORS.*

### *Association of Army and Navy Surgeons.*

This useful organization is increasing in numbers rapidly, and is destined, we hope, to accomplish great results. It represents not only the collected wisdom of the army and navy, but all gentlemen in the profession, who may desire it, can aid in effecting its commendable designs by being elected honorary members.

Hereafter, a regular report of the transactions of the Association will appear in our columns. Among many topics of interest now before it, we will especially mention—

*The statistics of secondary hemorrhage*—giving the period when it occurred, artery, and result.

*Chloroform.*

*Traumatic aneurism*—result of gun-shot wounds.

*Tetanus.*

Reports on important topics, from standing committees, add interest to the deliberations of this body, and we will especially note Surgeon Read's paper on the use of water dressings in gun-shot wounds, presented at a recent meeting. We cordially desire to see the medical staff and the profession at large united in harmonious action, each striving in its sphere to render the Association a brilliant and permanent success.

---

### *Resection at Hip Joint (Read's Case).*

The admirable wood cut, from a photograph of the patient whose surgical history we presented in our last issue, will give the reader a clear idea of the condition of the limb after resection of head of femur. This interesting subject, the importance of which can hardly be over-estimated, will be repeatedly a topic for consideration.

[Whilst we go to press, the following case of resection at hip-joint came under our notice. This soldier, an athletic young man, was rapidly walking up the street with the aid alone of his cane, a slight limp, indicating that he had been the subject of this formidable surgical procedure. We took from him the following sketch of his case:

W. F. Pumphrey, company "H," 1st Virginia infantry, was wounded *four times* at South Mountain—one wound, from a minie ball, fracturing the femur at its neck. He was carried to Frederick, Maryland, and, five weeks afterwards, Surgeon Hewitt, in charge of the United States Hospital at the Convent of Visitation, resected the head of the bone. The patient was taken to Baltimore and treated by Professor Miltenburger, who performed a second resection, taking away a portion of the shaft which had been left behind by Doctor Hewitt. After nine months' confinement to bed, this patient recovered with a shortening of less than *one inch*. He states that at first his leg was too long, but Professors N. R. Smith and Miltenburger applied the anterior splint and the limb gradually shortened.—Ed.]

---

*The Medical Department of the Confederate States Army—its relation to the other branches of the service, and the duties of its officers.*

Although the organization of the medical department is not as complete as it is believed it could have been, had the ideas and suggestions of its experienced presiding officer met with more favorable consideration, still, in view of the exactness with which its varied duties have been defined and systematized, it may be confidently asserted that, in the full performance of these duties by its members, the objects for which it was instituted have been, if not perfectly, yet, to a very great extent, satisfactorily accomplished.

So far as the definition of their duties is concerned, the officers of the department may be conveniently arranged in two classes:

The first: The more purely professional, in which a larger proportion of the duty is strictly of a professional nature, but in which administrative action enters as a valuable and necessary element, and of which it may indeed become, the more prominent feature.

The second: That class whose special duty is to provide and to dispense to the sick and wounded, medical and hospital supplies. With this class, professional knowledge as to the nature and necessity for these supplies under all circumstances, based upon the general education and experience of a medical officer, may, when compared with the also necessary possession on his part of some business capacity and a moderate degree of information as to the general mercantile laws regulating commercial economy, be affirmed to be of equal relative importance.

It is to the definition of the duties of these two classes of officers that attention will be directed, after brief allusion to the very delicate but responsible relations held by the medical department to all the other co-ordinate branches of the military service.

The strict maintenance of these relations, which are clearly laid down in the Army Regulations, and readily discernible upon its careful perusal, affects not only the department under consideration, but is intimately connected with the integrity and welfare of the service at large.

It is necessary that the medical officer should carefully avoid any encroachment upon the rights, position, or duties of the officers of other branches of the service, his chief and only ambition being the welfare of his patient and the attainment of the highest degree of excellence and merit in his own professional duties, without overstepping their proper limits.

Associated with these officers under all the varied and opposing conditions of ease and hardship, of calm and excitement, of security and peril, it behooves the medical officer, whilst studiously jealous of the rights and privileges of his position, to be ever fully sensible of the necessity for the strict observance of the requirements of regulations and general orders; respectful for and a co-operator in the enforcement of military discipline; careful to avoid a susceptibility to unintentional wrong, and ever mindful, in official intercourse, of the exercise of official respect, courtesy and forbearance.

In his duties to those whose physical sufferings and infirmities it is his special province to administer, often called upon to encounter risks of equal magnitude with those of the soldier, his cherished consolation and his chief incentive to renewed exertion should consist in the well-earned and enviable consciousness of a man of integrity in the full performance of an honorable philanthropic duty; and should it be required of him, as it may be upon the field and under the fire of the enemy, to risk his life, as did the illustrious Percy, to save that of another, he should always approach and enter upon this duty with that calmness, promptitude and decision so necessary to an advantageous application of his professional knowledge, and which the emergencies of the peculiar walk he has chosen have so great a tendency to develope. The unhesitating and manly performance of this duty, if not rewarded by the bestowal of deserved honor and position equal with that of the combatant of the line, must, at least in the hearts of all true patriots, be acknowledged by the universal accordance of praise and admiration.

The duties of the medical officers, as herein previously defined, may be further subdivided into those *administrative*, having direction of affairs, and those *executive*.

We propose, in our future issues, to sketch, somewhat in detail, the various and delicate functions of these two classes.

## Figures and Tables

**Figure f1:**